# Clinical picture and treatment implication in a child with Capgras syndrome: a case report

**DOI:** 10.1186/1752-1947-6-406

**Published:** 2012-11-27

**Authors:** Luigi Mazzone, Marco Armando, Franco De Crescenzo, Francesco Demaria, Giovanni Valeri, Stefano Vicari

**Affiliations:** 1 Child Neuropsychiatry Unit, Department of Neuroscience, I.R.C.C.S. Children’s Hospital Bambino Gesù, Rome, Italy

**Keywords:** Capgras, Children, Risperidone, Selective serotonin reuptake inhibitors

## Abstract

**Introduction:**

Capgras syndrome is a delusional misidentification syndrome characterized by the patient’s belief that his or her relatives have been replaced by impostors.

**Case presentation:**

Here we describe the clinical picture and the therapeutic approach to an 11-year-old Caucasian girl with Capgras syndrome. A complete psychopathological assessment was conducted during the acute phase, at one month, two months and six months since diagnosis.

**Conclusion:**

Subsequent follow-up evaluations in this patient allowed us to detect improvements in the psychotic symptoms following treatment with risperidone and selective serotonin reuptake inhibitors, suggesting that this combined therapy may significantly improve the clinical outcome in patients who have Capgras syndrome.

## Introduction

Capgras syndrome [1] is a monothematic delusion characterized by the patient’s belief that his or her relatives have been replaced by impostors who have a close resemblance to the originals [2]. Previous reports in adults referred to this syndrome as a secondary manifestation of neurodegenerative syndromes, such as Alzheimer disease or patients showing brain damage, especially in the right frontal or in the left temporal lobe [3,4]. Indeed, other studies described a similar clinical picture in patients with an ‘at risk’ mental state or schizophrenia and borderline cognitive level [5,6]. Gender comparison reveals a higher prevalence in females than in males, with a 2:1 ratio. However, only a few reports have described this syndrome in male patients during childhood [2,7] and, to our knowledge, only one case has been reported for a girl: a 12-year-old girl who had been hospitalized for attacks of abdominal pain [8]. Neuroleptic drugs are widely used for the treatment of psychotic symptoms in children and adolescents, for whom they are considered currently the best option [9]. Neuroleptic drugs have also been used in patients with Capgras syndrome [10], although without a complete remission of the positive symptoms. A previous study [11] showed a positive outcome in a patient with Capgras syndrome after treatment with an antidepressant drug (mirtazapine). In the present report, we outline the clinical picture of an 11-year-old girl diagnosed with Capgras syndrome, showing signs of psychotic depression and the therapeutic approach that was chosen for her.

## Case presentation

An 11-year-old Caucasian girl with acute delusions and aggressive behavior was admitted to our acute in-patient Child Psychiatry Unit.

This patient was the first child of non-consanguineous parents, born at term by Caesarean section with a birth weight of 3,450kg. The neonatal period was regular, with maternal nursing. Autonomous walking occurred at 14 months and the first words were spoken at 12 months with a subsequent normal language development.

The patient showed normal learning abilities and good social skills before the onset of the psychiatric symptoms, even if her parents described her as ‘shy’ and with low social initiative; moreover, they reported behavioral modifications with a reduction of interests and social withdrawal a few months before the beginning of delusional symptoms. Her emotional relationship toward her parents was reserved and unstable.

A month before admission to the in-patient unit the girl started to develop suspicious thoughts with obsessive features concerning the possibility of being harmed by her parents, accompanied by a high level of anxiety. These symptoms soon worsened and the patient was referred to the Emergency Service for a clinical picture characterized by auditory hallucinations and persecutory delusions towards her parents. In detail, she believed that, after a short trip, her parents had been replaced by impostors and that the ‘new’ persons replacing them were planning to poison and kill her. At this point she showed a depressive mood with frequent sadness and crying, loss of energy and difficulties in sleeping and concentrating. Delusion was not extended to any other person or inanimate object, and was not associated with reduplicative paramnesia. Her parents showed at the time of admission a sense of guilt and a depressive mood with unsupportive interactions with their child. At the following visit they revealed an emotional feeling characterized by difficulties in caring for their child, frustration, and anger toward the child herself. Nevertheless, their overall social skills in interaction and communication were within the normal range [12].

A diagnosis of ‘Delusional Episode’ based on the *Diagnostic and Statistical Manual of Mental Disorders* (4th edition) was performed using Schedule for Affective Disorders and Schizophrenia for School-Age Children. The qualitative pattern of the delusion, concerning the identity of her parents despite still recognizing the semblance of their body, is typical of Capgras syndrome, which is the best known among delusional misidentification syndromes [2]. The girl was normal at both physical and neurological examination. The electroencephalography was also normal, whereas the magnetic resonance imaging (MRI) showed a cerebellar arachnoid cyst of non-pathological relevance. The results of other diagnostic tests including electrocardiography, sequential multiple analysis comprehensive metabolic panel, complete blood count, serum electrophoresis and thyroid function, were within normal range.

A complete psychopathological assessment was conducted at the time of admission (T0), and after one month (T1), three months (T2) and six months (T3). Between T0 and T1 the patient was treated with risperidone 3mg per day, and between T2 and T3 50mg per day of sertraline was added to the risperidone.

At T0 (no medication) the girl showed a full-scale intelligence quotient of 82 using the Wechsler Intelligence Scale for Children-III (Verbal Scale score: 86; Performance Scale score: 82), a Children’s Global Assessment Scale score of 39, high levels of depressive symptoms with a Children’s Depression Inventory score of 27, and a score of 71 on the Multidimensional Anxiety Scale for Children for anxiety symptoms. The Positive and Negative Symptom Scale (PANSS) for schizophrenia revealed a positive subscale score of 22, a negative subscale score of 20, a general psychopathology subscale score of 41 and a total score of 83. Finally, the girl scored 7 on the Clinical Global Impression-Severity (CGI-S) scale.

At T1 (after one month of treatment with risperidone, 3mg per day), a reduction was observed in the positive symptoms, including auditory hallucinations, albeit still relevant. By contrast, the CGI-S showed only a minimal improvement and no reduction in the depressive symptoms was detected (see Table 1). Due to the modest improvement of the clinical picture and to the stable high levels of depressive symptoms and obsessive thoughts, clinicians decided to introduce an antidepressant in association with risperidone: the choice was sertraline (50mg per day).

**Table 1:** Longitudinal psychopathological assessment

	**T0**	**T1**	**T2**	**T3**
	***At admission***	***After one month of treatment with 3mg risperidone***	***Two months after addition of sertraline***	***After six months of treatment***
CGAS	39	40	50	58
CGI-Severity	7	6	3	2
CGI-Improvement	N/A	3	1	1
PANSS Positive	22	19	12	9
CDI	27	27	17	15
MASC Total score	71	70	65	52

CDI, Children’s Depression Inventory; CGAS, Children’s Global Assessment Scale; CGI, Clinical Global Impression; MASC, Multidimensional Anxiety Scale for Children; PANSS, Positive and Negative Syndrome Scale.

At T2, after two months of combined treatment, a significant improvement was observed in all the domains analyzed, with a full remission of the clinically relevant psychotic symptoms, as assessed in the clinical setting and by direct interviews. Finally, a significant reduction in the severity of symptoms was also detected after six months (T3) of treatment with risperidone and sertraline: the patient started to recognize her parents and the monothematic delusion characterized by her belief of parents being replaced by impostors disappeared. Moreover, the PANSS scores for schizophrenia also showed a progressive reduction over time: from 22 at admission to 19, 12 and 9 at T1, T2, and T3, respectively (see Table 1).

## Discussion

In this report we describe a young girl showing psychotic symptoms that can be ascribed to a clinical picture of Capgras syndrome. The etiopathogenesis of this syndrome seems to involve organic factors and a difficulty in processing facial recognition, particularly towards a family member [13]. As a consequence, patients who have this syndrome are often characterized by the absence of emotional feelings towards persons of their own family [14].

In the literature, difficulty in facial recognition is often referred to as prosopagnosia. However, people with prosopagnosia can recognize an individual when listening to his or her voice and under this auditory perception they do not consider the possibility of the other being an imposter [15]. An overlapping between Capgras syndrome and prosopagnosia has been proposed and some authors have hypothesized this syndrome as the ‘mirror image’ of prosopagnosia [2,16]. Neuroanatomical findings showed that the ventral pathway of explicit facial recognition is altered in prosopagnosia, whereas the dorsal pathway, responsible for the emotional processing of facial recognition, seems to be conserved. By contrast, in patients with Capgras syndrome the ventral pathway remains intact, whereas the dorsal pathway is impaired, providing a possible explanation for the altered emotional responses [2].

A critical point of our report is the positive outcome in response to the combination of an antipsychotic medication (risperidone) and a selective serotonin reuptake inhibitor (SSRI). Literature documents the efficacy of risperidone for the treatment of psychotic symptoms in children [17,18], supporting the rationale for our intervention. However, the observed increase in depressive and obsessive symptoms despite a partial remission of the symptomatology after one month of risperidone therapy provided the rationale for the concomitant administration of a SSRI. Besides there is scant available evidence regarding the association between antipsychotic drugs and SSRIs in children [19]; in this patient this association led to an improvement in both depressive and obsessive symptoms as well as to a complete remission of the overall Capgras clinical picture. Of note, it has been shown that when this syndrome occurs in a depressive setting its prognosis follows the comparatively better prognosis of depression as compared to that of schizophrenia [20,21]. Therefore, it is possible that the depressive component in the symptomatology of this patient has contributed to the better outcome of the overall clinical picture.

Despite the fact that risperidone and SSRIs have been shown to cause severe adverse effects including marked weight gain, sedation, and risk of extrapyramidal symptoms, none of these side effects were observed in our patient.

## Conclusion

Capgras syndrome is very rare in childhood. Our case suggests that a combined treatment of an antipsychotic medication plus a SSRI can lead to a good clinical outcome with a remission of the psychotic symptoms.

## Consent

Written informed consent was obtained from the patient’s legal guardian for publication of this case report and accompanying images. A copy of the written consent is available for review by the Editor-in-Chief of this journal.

## Competing interests

The authors declare that they have no competing interests.

## Authors’ contributions

LM and MA reviewed the literature, pulled all the information together, wrote the manuscript and contributed equally to the paper; FD and GV followed the patients and collected the data. FDC reviewed the literature; SV contributed to theoretical interpretation and final proofreading. Each author read and approved the final version of the manuscript.
